# A Highly Sensitive Telomerase Activity Assay that Eliminates False-Negative Results Caused by PCR Inhibitors

**DOI:** 10.3390/molecules181011751

**Published:** 2013-09-25

**Authors:** Hidenobu Yaku, Takashi Murashima, Daisuke Miyoshi, Naoki Sugimoto

**Affiliations:** 1 Advanced Technology Research Laboratories, Panasonic Corporation, 3-4 Hikaridai, Seika-cho, Soraku-gun, Kyoto 619-0237, Japan; E-Mail: yaku.hidenobu@jp.panasonic.com; 2 Frontier Institute for Biomolecular Engineering Research (FIBER), Konan University, 7-1-20 Minatojima-minamimachi, Chuo-ku, Kobe 650-0047, Japan; E-Mail: murasima@konan-u.ac.jp; 3 Faculty of Frontiers of Innovative Research in Science and Technology (FIRST), Konan University, 7-1-20 Minatojima-minamimachi, Chuo-ku, Kobe 650-0047, Japan

**Keywords:** telomerase, PCR inhibitor, magnetic beads, asymmetric PCR, cycling probe technology

## Abstract

An assay for telomerase activity based on asymmetric polymerase chain reaction (A-PCR) on magnetic beads (MBs) and subsequent application of cycling probe technology (CPT) is described. In this assay, the telomerase reaction products are immobilized on MBs, which are then washed to remove PCR inhibitors that are commonly found in clinical samples. The guanine-rich sequences (5'-(TTAGGG)*n*-3') of the telomerase reaction products are then preferentially amplified by A-PCR, and the amplified products are subsequently detected *via* CPT, where a probe RNA with a fluorophore at the 5' end and a quencher at the 3' end is hydrolyzed by RNase H in the presence of the target DNA. The catalyst-mediated cleavage of the probe RNA enhances fluorescence from the 5' end of the probe. The assay allowed us to successfully detect HeLa cells selectively over normal human dermal fibroblast (NHDF) cells. Importantly, this selectivity produced identical results with regard to detection of HeLa cells in the absence and presence of excess NHDF cells; therefore, this assay can be used for practical clinical applications. The lower limit of detection for HeLa cells was 50 cells, which is lower than that achieved with a conventional telomeric repeat amplification protocol assay. Our assay also eliminated false-negative results caused by PCR inhibitors. Furthermore, we show that this assay is appropriate for screening among G-quadruplex ligands to find those that inhibit telomerase activity.

## 1. Introduction

Telomerase is responsible for the elongation of telomere DNA, which comprises repeated units of guanine rich (G-rich) sequences (e.g., 5'-TTAGGG-3') at chromosome ends [1,2,3]. In 85%–90% of human tumor cells, telomerase activity is abnormally high and plays a key role in immortal cell proliferation [4,5,6]. Thus, telomerase activity may be a promising marker for cancer diagnosis [7,8]. Several studies have shown an association between telomerase activity and a number of prognostic and clinicopathological features in colorectal cancer [9,10,11,12,13,14,15]. To use telomerase activity as a diagnostic marker, an accurate assay for telomerase activity is required.

Telomeric repeat amplification protocol (TRAP) assay is a commonly used conventional assay for telomerase activity; in TRAP, a telomerase reaction is followed by polymerase chain reaction (PCR) [6]. TRAP assay can detect telomerase activity with high sensitivity, but are susceptible to false-negative results due to PCR inhibition by biomolecules such as bile salt, hemoglobin, heparin, and lactoferrin [16]. Thus, some research groups have developed other telomerase assays based on various sensors or methodologies, such as optical fibers [17], magnetic resonance readers [18], magneto-mechanical detectors [19], ion sensitive field effect transistor (ISFET) [20], electrochemical detection [21], photonic microring devices [22], or surface plasmon resonance [20,23], but in general such assays do not utilize any signal amplification processes like PCR and were therefore less sensitive than TRAP assay. Other groups proposed telomerase assays with novel signal amplification processes involving enzymes [24,25,26,27], DNAzymes [28,29,30], and nanoparticles [31,32,33,34] instead of PCR. Although some of these assays with novel amplification reactions detected telomerase activity with high sensitivity, the enzymes used for catalysis-based amplification may also be inhibited by components in clinical samples.

Here, for sensitive detection of telomerase activity that avoids false-negative results, we report a telomerase assay based on asymmetric PCR (A-PCR) [35,36] on magnetic beads (MBs) and subsequent application of cycling probe technology (CPT) [37] involving a probe RNA and RNase H. In principle with this assay, telomerase reactions can be run with crude clinical samples, and because the reaction products are attached to MBs, they can be washed to remove contaminants from clinical samples including PCR inhibitors. This washing process demonstrably reduces the false-negative results caused by PCR inhibition. The G-rich sequences [5'-(TTAGGG)n-3'] of the telomerase reaction products are then preferentially amplified by A-PCR and are detected by CPT. In CPT, a probe RNA with a fluorophore at the 5' end and a quencher at the 3' end hybridized with target G-rich sequences; hybridized probe RNA is the preferentially hydrolyzed by RNase H. The hydrolysis of the probe separates the fluorophore from the quencher, which results in enhancement of the fluorescent signal. In addition, each reaction—including the hybridization of the probe RNA with the G-rich sequences and the hydrolysis of the hybridized probe RNA by RNase H—occurs repeatedly; these repetitive processes ultimately lead to signal amplification. CPT with probe RNA (5'-CCCUAACCC-3') detected a model sequence of telomerase reaction product (MSTP) comprising 5'-(GGGTTA)_16_-3'; the lower limit of detection was 100 amol; therefore, CPT was two orders of magnitude more sensitive than conventional gel electrophoresis analysis. Furthermore, we could use this telomerase assay based on a combination of A-PCR on MBs and CPT with a probe RNA to specifically and sensitively detect HeLa cells among cell populations comprising predominantly normal human dermal fibroblast (NHDF) cells. The detection limit of this assay for HeLa cells was 50 cells, making this assay therefore more sensitive than the common TRAP assay. Moreover, we show that the assay eliminated false-negative results caused by common components of clinical samples that inhibit PCR such as bile salt, hemoglobin, and heparin. Our results demonstrated that this assay is practical for detecting cancer cells in clinical samples.

## 2. Results and Discussion

### 2.1. Detection Principle

**Figure 1:**
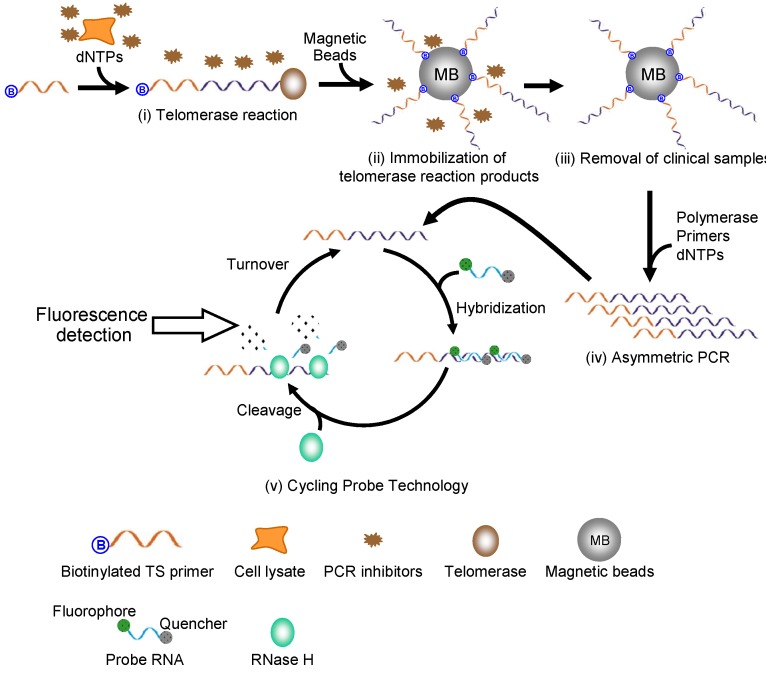
Strategy for the telomerase assay based on A-PCR on MBs and CPT: (**i**) Telomerase in crude clinical extract elongates the telomere DNA sequence from biotinylated TS primer; (**ii**) Telomerase reaction products are immobilized on MBs *via* interaction between biotin and streptavidin; (**iii**) MBs coated with the telomerase products are washed to remove PCR inhibitors; (**iv**) The G-rich sequences of the telomerase products are preferentially amplified *via* A-PCR; (**v**) Amplified G-rich sequences are detected by CPT.

Figure 1 depicts the principle of the telomerase assay described here. In principle, the assay includes the following steps: (i) Telomerase elongates the telomere DNA sequence *via* a biotinylated TS primer (Table 1), which serves as a substrate for telomerase elongation; (ii) The biotin-labelled telomerase reaction products are immobilized on streptavidin-coated MBs *via* interaction between biotin and streptavidin; (iii) MBs coated with telomerase products are washed to remove sample contaminants including PCR inhibitors; (iv) The G-rich sequences of the telomerase products are preferentially amplified by A-PCR; (v) Amplified G-rich sequences are then detected *via* CPT. In CPT, a probe RNA with a fluorophore (FITC) and a quencher (Dabcyl) at the 5' end and 3' end, respectively, hybridizes with the G-rich sequences. The hybridized probe RNA is hydrolyzed by RNase H, which recognizes RNA/DNA duplex and selectively hydrolyzes RNA in heteroduplexes. Before hydrolysis, fluorescence from FITC is quenched by fluorescence resonance energy transfer (FRET) due to proximity between FITC and Dabcyl. However, the hydrolysis of the probe RNA separates FITC from Dabcyl, which results in enhancement of the FITC fluorescence. Additionally, each reaction—including the hybridization of the probe RNA with the telomerase reaction products and the hydrolysis of the hybridized probe RNA by RNase H—occur iteratively, which leads to a catalytic amplification of FITC signal. Importantly, in principle, the false-negative results caused by PCR inhibitors should be completely avoided, and the combined application of A-PCR and CPT should lead to highly sensitive and selective detection of telomerase activity.

### 2.2. Design of the Probe RNA

The sequence and design of the probe RNA used for CPT is responsible for the sensitivity of this assay. Reducing the length of the probe should show lower background signals because FITC should be in closer proximity to Dabcyl; however, affinity between the probe and telomerase products should be lower with shorter probes. Conversely, longer probes should exhibit higher affinity for telomerase products and higher background signal. To optimize the probe RNA, four probes with FITC and Dabcyl at the 5' end and 3' end, respectively, were designed; the probes differed from one another in length and in sequence (Table 1).

**Table 1:** RNA oligonucleotides used in this study.

Name	Sequence	Modification
Probe 1 ^a^	5'-CCCUAA-3'	5'-FITC/3'-Dabcyl
Probe 2 ^a^	5'-CCCUAACCC-3'	5'-FITC/3'-Dabcyl
Probe 3 ^a^	5'-CUAACCCUAAC-3'	5'-FITC/3'-Dabcyl
Probe 4 ^a^	5'-CCCUAACCCUAACCC-3'	5'-FITC/3'-Dabcyl
MSTP ^b^	5'-(GGGTTA)_16_-3'	none
TS primer ^c^	5'-AATCCGTCGAGCAGAGTT-3'	none
Biotinylated TS primer ^d^	5'-AATCCGTCGAGCAGAGTT-3'	5'-biotinylation
CX-ext ^e^	5'-GTGCCCTTACCCTTACCCTTACCCTAA-3'	none

*^a^* Probes are FRET-modified complementary RNAs to telomere sequence; *^b^* MSTP is a model sequence of a telomerase product; *^c^* TS primer is a substrate DNA for telomerase and a forward primer for PCR amplification of telomerase reaction products; *^d^* Biotinylated TS primer is a TS primer with a 5' biotin moiety for immobilization on streptavidin-coated magnetic beads; *^e^* CX-ext is a reverse primer for PCR amplification of telomerase reaction products.

**Figure 2:**
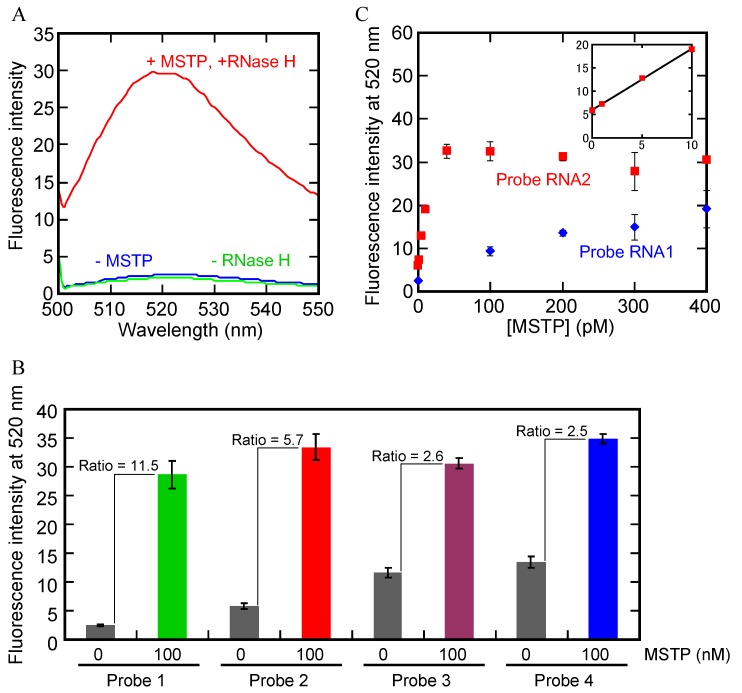
(**A**) Fluorescence spectra with probe 1 in the absence or presence of RNase H, MSTP or both; (**B**) Fluorescence at 520 nm with probes 1–4 in the absence or presence of MSTP. Each value is the average calculated from the three replicate data sets and each error bar represents the standard deviation; (**C**) Plots of fluorescence at 520 nm with probe 1 or probe 2 *vs.* MSTP concentration. Each data pint is the average calculated from the three replicate data sets and each error bar represents the standard deviation.

First, we carried out the RNase H reaction separately for each probe with 100 nM of probe in the absence or the presence of 100 nM MSTP (Table 1) at 37 °C for 30 min. For probe 1, an obvious peak of fluorescence with a maximum intensity around 520 nm was observed in the presence of both RNase H and MSTP (Figure 2A). In contrast, in the absence of MSTP, the peak at 520 nm was clearly smaller, and the fluorescence at 520 nm was about 18 times lower than that in the presence of both RNase H and MSTP (Figure 2A). Additionally, the fluorescence in the absence of RNase H was almost identical to that in the absence of MSTP. These results indicate that probe 1 hybridized with MSTP and that as expected RNase H hydrolyzed only the hybridized probe. Each other probe (probes 2, 3, and 4) also exhibited more fluorescence in the presence of MSTP than that in the absence of MSTP (Figure 2B). However, the signal/background ratio values of probe 3 (2.6) and probe 4 (2.5) were less than those of probe 1 (11.5) and probe 2 (5.7). This difference occurred because, in the absence of MSTP, longer probes (probes 3 and 4) resulted in higher background fluorescence than did shorter probes (probes 1 and 2, Figure 2B). 

**Figure 3:**
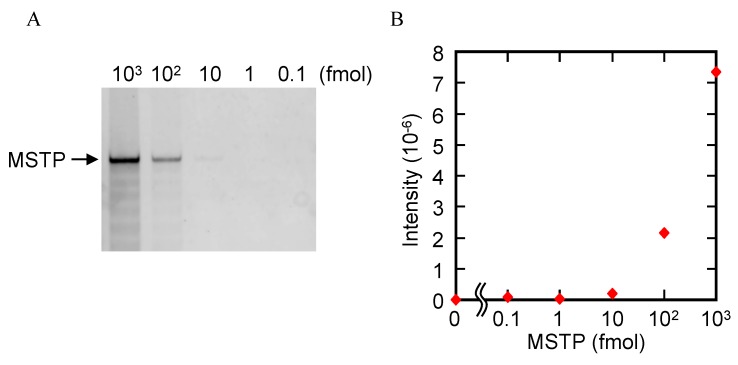
(**A**) Electrophoresis results of five different amounts of MSTP. (**B**) Relationship between band intensity of and amount of MSTP.

We further investigated the sensitivities of probes 1 and 2 by carrying out the RNase H reaction with different concentrations (0–400 pM) of MSTP. The fluorescence at 520 nm of probe 1 decreased gradually as the concentration of MSTP decreased (Figure 2C). However, the fluorescence from probe 2 with concentrations of MSTP between 80–400 pM were similar to each other and higher than those from probe 1; the fluorescence from probe 2 did decrease with decreases in MSTP concentration from 0–60 pM MSTP (Figure 2C). Furthermore, we found a linear relationship between MSTP concentration and probe 2 fluorescence; the correlation equation for this relationship was probe 2 fluorescence at 520 nm = 6.01 + 1.31 × (MSTP concentration (pM)) (*R*^2^ = 0.9986) in the range of 0–10 pM (inset in Figure 2C). Based on this linear relationship, the limit of detection with probe 2 was calculated to be 0.99 pM using the following Equation (1):

DL = 3.3 δ/S
(1)

where DL is the detection limit; δ is the standard deviation of the blank sample; and S is the slope of the correlation equation obtained by the linear relationship. The reaction volume was 100 μL; therefore, CPT with probe 2 should be detected MSTP at concentrations as low as 100 amol. This concentration is two orders of magnitude lower than that detectable with conventional gel electrophoresis analysis (Figure 3). These results showed that probe 2 is suitable for CPT and detection of telomerase reaction products.

### 2.3. Inhibition of Degradation of Telomerase Reaction Products by Decoy DNA

**Figure 4:**
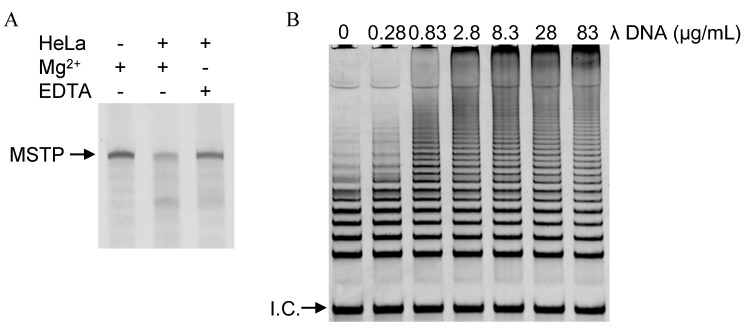
(**A**) Electrophoresis of MSTP-based telomerase reaction products in the absence or presence of HeLa cells lysates, Mg^2+^, and EDTA; (**B**) Electrophoresis of PCR-amplified telomerase reaction products synthesized in the presence of various concentrations of λ DNA.

Cell lysate used without any prior purification contains various intracellular biomolecules, including DNase, that can degrade the products of telomerase reactions. Therefore, contaminants such as DNase can lead to false-negative results from telomerase assays. To confirm that such problems occur, MSTP with or without HeLa cells lysate was kept in a buffer containing 50 mM MES-LiOH (pH 7.0) and 4 mM MgCl_2_ at 37 °C for 60 min, and the degradation of MSTP was analyzed *via* gel electrophoresis. In the absence of HeLa cell lysate, a band corresponding to MSTP was observed (Figure 4A); however, addition of HeLa cells lysate reduced the MSTP band. Moreover, when EDTA was used instead of Mg^2+^, addition of HeLa cells lysate did not reduce MSTP band (Figure 4A). These results indicated that DNase in cell lysate can degrade telomerase reaction products. To prevent such degradation, we reasoned that addition of excess decoy DNA could prevent DNase from degrading the telomerase reaction products because the decoy DNA should serve as a competitive substrate for DNase. 

This assumption was confirmed by performing telomerase reaction with HeLa cells lysate in the absence or presence of various concentrations of λ DNA as the decoy DNA. The telomerase reaction mixtures were diluted 50-fold to reduce the influence of the decoy λ DNA on subsequent PCR; the telomerase reaction products were then amplified *via* PCR. PCR products were monitored *via* gel electrophoresis (Figure 4B). In the absence of λ DNA, long telomerase products were not abundant (Figure 4B), indicating that DNase in HeLa cells lysate degraded the telomerase reaction products. However, long telomerase products were observed telomerase reactions containing λ DNA, and the abundance of long products increase with increasing concentrations of λ DNA (Figure 4B). These results indicate that λ DNA efficiently inhibited DNase from degrading the telomerase reaction products by serving as a competitive substrate for DNase. Thus, addition of excess decoy DNA is a useful, easy, and cost-effective method for avoiding false-negative results caused by DNase.

### 2.4. Optimization of Primers Concentrations for A-PCR

**Figure 5:**
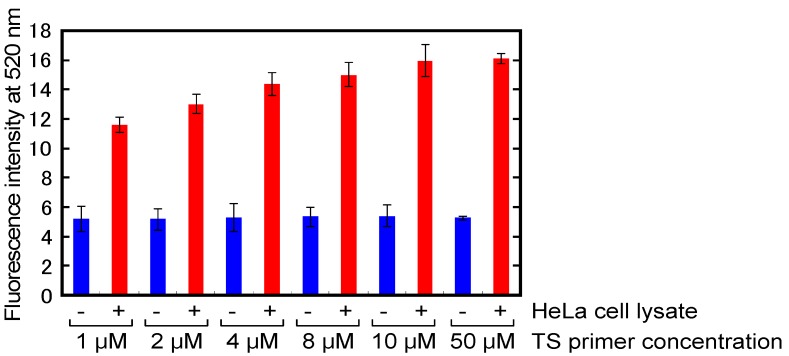
Fluorescence at 520 nm of probe 2 resulting from CPT with telomerase reaction products amplified *via* A-PCR using five different concentrations of TS primer. Each value is the average calculated from the three replicate data sets and each error bar represents the standard deviation.

In principle, we use CPT with probe RNA and RNase H to achieve high sensitivity, and target DNA for CPT should be single-stranded DNA. A-PCR is appropriate for generating the CPT target DNA because the predominant product of A-PCR is single-stranded DNA extended from a primer that is present in much higher concentrations than its partner primer. To optimize the concentration ratio of forward/reverse primers, telomerase reaction products in the absence or presence of 200 HeLa cells lysate were amplified by A-PCR with 1 μM to 50 μM TS primer and 1 μM CX-ext primer [38,39] (Table 1) as the forward and reverse primers, respectively. The CX-ext primer, 5'-GTGCCCTTACCCTTACCCTTACCCTAA-3', is complementary to four telomere repeats but contains a single base mismatch at the same position in each of three of the telomere repeats, and has three additional non-complementary nucleotides at its 5' end [38,39]. Therefore, the CX-ext primer functions as a reverse primer that reduces PCR-associated artifacts [38,39]. CPT with probe 2 was used to detect the products amplified *via* A-PCR. In the absence of HeLa cell lysate, the fluorescence from probe 2 at 520 nm did not depend on the concentration of TS primer and was identical to the fluorescence from probe 2 without MSTP in Figure 2 (Figure 5). These results indicate that even highly concentrated TS primer did not generate any artifacts when paired with CX-ext primer, even though highly concentrated primers often cause PCR-associated artifacts. On the contrary, in the presence of HeLa cells lysate, the fluorescence from probe 2 with each concentration of TS primer was higher than that in the absence of HeLa cells lysate (Figure 5). In addition, increases in the primer concentration up to 10 μM TS primer enhanced the fluorescence at 520 nm, and the fluorescence with 50 μM TS primer was almost the same as that with 10 μM TS primer. These results indicated that the amount of single-stranded DNA that comprised telomere repeats increased as the concentration of TS primer increased up to 10 μM. Thus, 10 μM TS primer and 1 μM CX-ext primer were used in each subsequent assay.

### 2.5. Detection of Telomerase Activity in Cells Lysate

**Figure 6:**
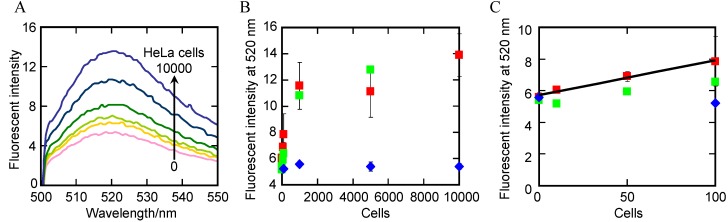
(**A**) Fluorescence spectra results from the A-PCR/CPT assays run with lysate with various concentrations of HeLa cells; (**B**,**C**) Plots of fluorescence intensity at 520 nm *vs.* the number of cells (red square: HeLa cells, blue diamond: NHDF cells, green square: HeLa cells in the presence of 5,000 NHDF cells). Each data point for HeLa cells (red square) and NHDF cells (blue diamond) is the average calculated from the three replicate data sets, and each error bar represents the standard deviation.

Based on the results described above, telomerase activity in HeLa cells lysate could be detected as follows: (i) Telomerase reaction carried out in the presence of λ DNA at 37 °C for 60 min; (ii) Telomerase reaction products immobilized on MBs at 25 °C for 30 min, and then washed with clean buffer; (iii) Telomerase reaction products on MBs amplified over 30 cycles of A-PCR with 10 μM TS primer and 1 μM CX-ext primer; (iv) A-PCR products are detected by CPT with probe 2. We carried out these procedures with various numbers of HeLa cells (Figure 6A). The fluorescence at 520 nm increased as a function of the number of HeLa cells (red square in Figure 6B). We also found a linear relationship between fluorescence intensity and HeLa cell number; the correlation equation was fluorescence intensity = 5.71 + 0.022 × (number of HeLa cell) (*R*^2^ = 0.984) within the range of 0–100 cells (Figure 6C). Based on this linear relationship, the limit of detection was calculated to be 50 cells using the above equation (1). These results indicate that the difference between the signal from 50 HeLa cells and that from no HeLa cells was statistically significant and the sensitivity of the present assay is higher than that of the common TRAP assay [40,41]. In contrast, 100, 1000, 5000, or 10,000 NHDF cells, which have no telomerase activity, caused no fluorescence when used in our assay ((the fluorescent intensity with NHDF cells)–(the fluorescence intensity with no cells) < 0.01) (blue diamonds in Figure 6B,C). These results demonstrate that the fluorescence intensity depends on the telomerase activity. In addition, the fluorescence intensity when probing samples from 100–10,000 NHDF cells was almost the same as that when probing samples lacking cells, although RNA is very unstable especially in clinical samples. This lack of background probably resulted because the probe RNA was added into each CPT reaction mixture after most components of the cell extracts were separated from the telomerase reaction products. Thus, the probe RNA was not degraded by irrelevant components of the cell extracts, thereby avoiding false-positive results. These results indicate that this assay should be useful for detecting cancer cells with high sensitivity and specificity. Importantly, we also found that, due to the high sensitivity, the fluorescence corresponding to the number of HeLa cells in the presence of 5,000 NHDF cells (green square in Figure 6B) was identical to that in the absence of NHDF cells. These results indicate that the assay is useful for practical for clinical samples, in which cancer cells should be selectively detected in the presence of a large excess of normal cells.

### 2.6. Eliminating False-Negative Results Caused by PCR Inhibitors

**Figure 7:**
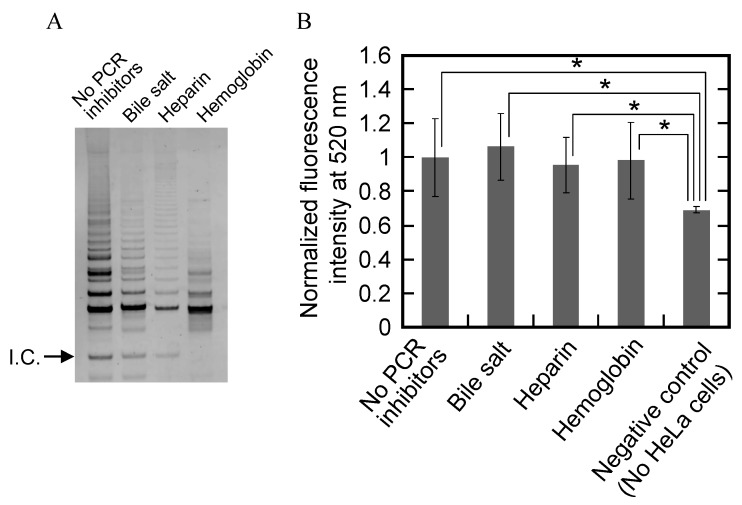
(**A**) Electrophoresis results from the one-step TRAP assay in the absence or presence of individual PCR inhibitors; (**B**) Fluorescence intensity results from the A-PCR/CPT assay with or without individual PCR inhibitors.Each value is the average calculated from the five replicate data sets and each error bar represents the standard deviation. * *p* < 0.05.

PCR inhibitors that exist in cells quantitatively reduce PCR amplification. The previous study showed that bile salt, heparin, and hemoglobin inhibited PCR at ≤1 mg/mL, ≤1 IU/mL, and ≤1 mg/mL order or less, respectively [42]. The washing process on MBs in the present assay should eliminate the false-negative results caused by such inhibitors. Therefore, we investigated effects of these PCR inhibitors—bile salt (1 mg/mL), heparin (2 IU/mL), and hemoglobin (1 mg/mL)—on the common one-step TRAP assay and our A-PCR/CPT assay; both assays were run with HeLa cells lysate. In the one-step TRAP assay, all reagents for telomerase reaction, PCR, and the PCR inhibitors are included together in the reaction solution during the telomerase reaction and the PCR. The gel resulting from the one-step TRAP assay showed that ladder-like bands corresponding to the TRAP assay products and an internal control band corresponding to PCR products from a 36-bp template were evident in the absence of PCR inhibitors (lane 1 in Figure 7A). However, each inhibitor drastically reduced the amount of TRAP assay products and of the internal control PCR products (lanes 2–4 in Figure 7A). These results indicate that a reduction of TRAP assay products was caused, at least in part, by PCR inhibitors. In contract, the fluorescence intensity results from the A-PCR/CPT assay showed that the fluorescence at 520 nm was similar regardless of the presence or absence of bile salt, hemoglobin, or heparin, and their fluorescence was significantly larger than that from the negative control without HeLa cells (*p* value < 0.05) (Figure 7B). These results indicate that the A-PCR/CPT assay, unlike the common one-step TRAP assay, can detect telomerase activity and simultaneously avoid the false-negative results caused by PCR inhibitors, including bile salt, heparin, and hemoglobin. Thus, the present assay should detect cancer cells with higher sensitivity than the common one-step TRAP assay in the presence of PCR inhibitors.

### 2.7. Evaluation of Telomerase Inhibitory Effects of Anionic Phthalocyanine

**Figure 8:**
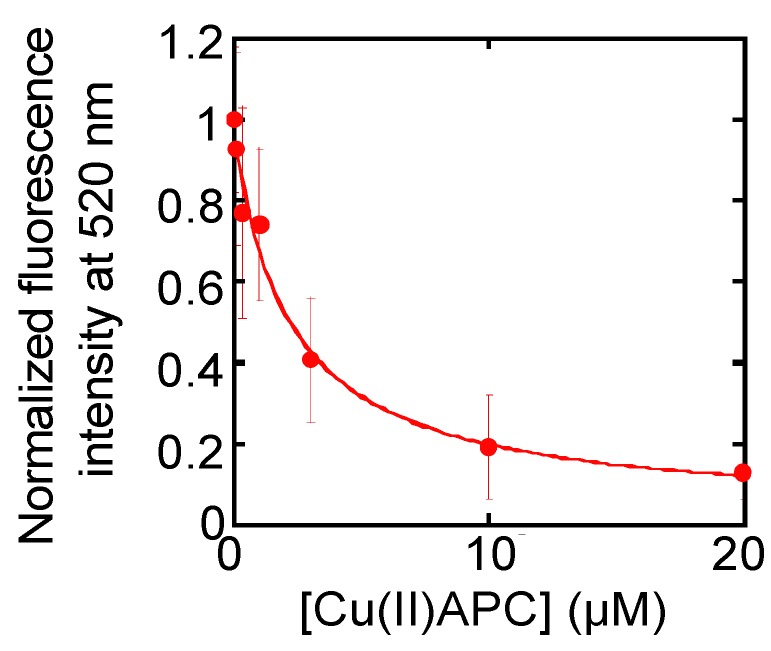
Fluorescence intensity results from the A-PCR/CPT assay in the presence of various concentration of Cu(II)APC. Each data point is the average calculated from the three replicate data sets and each error bar represents the standard deviation.

Telomere DNA can form four-stranded DNA called G-quadruplex [43]. G-quadruplex does not serve as a substrate DNA for telomerase [44]; therefore, G-quadruplex ligands that inhibit telomerase activity are promising as anticancer drugs [45]. For development of G-quadruplex ligands, a quantitative evaluation of the telomerase inhibitory effects of such ligands is required. Reportedly, the common one-step TRAP assay is not appropriate for such evaluation because G-quadruplex ligands can also inhibit PCR and lead to false-negative results [46]. In contrast, the A-PCR/CPT assay can accurately evaluate the inhibitory effects of these ligands because this assay should eliminate PCR inhibition, as demonstrated above. To assess this supposition, we used the A-PCR/CPT assay to evaluate the effects of a G-quadruplex ligand on telomerase activity. For this purpose, the A-PCR/CPT assay was carried out with lysate from 1,000 HeLa cells and in the presence of 0–20 μM copper(II) phthalocyanine 3,4',4'',4'''-tetrasulfonic acid, tetrasodium salt (Cu(II)APC), which binds to G-quadruplex DNA specifically over dsDNA and inhibits telomerase activity [47,48,49,50]. The normalized fluorescence intensity at 520 nm was plotted *vs.* the concentration of Cu(II)APC, and the fluorescence decreased as a function of the concentration of Cu(II)APC (Figure 8). This result indicates that Cu(II)APC inhibited telomerase. Based on the plot, the IC50 value of Cu(II)APC for telomerase activity was estimated to be 2.2 ± 0.5 μM. The value is identical to the IC50 value (1.2 or 1.4 μM) obtained by an improved TRAP assay [47,50], which reduced the influence of Cu(II)APC on PCR by diluting the telomerase reaction products with Cu(II)APC. Therefore, the A-PCR/CPT assay can be used to screen for G-quadruplex ligands that inhibit telomerase activity.

## 3. Experimental

### 3.1. Materials and Reagents

All deoxyribo-oligonucleotides were high-performance liquid chromatography (HPLC) purification-grade deoxyribo-oligonucleotides, and were purchased from Tsukuba Oligo Service Co., Ltd., (Ibaraki, Japan), or were provided in a TRAPEZE telomerase detection kit from EMD Millipore Corporation (Billerica, MA, USA). λ DNA was purchased from Takara Bio Inc. (Shiga, Japan). All ribo-oligonucleotides were HPLC purification-grade ribo-oligonucleotides, and were purchased from Tsukuba Oligo Service Co., Ltd. HeLa and NHDF cells were provided in TRAPEZE telomerase detection kits and purchased from Toyobo Co., Ltd. (Osaka, Japan), respectively. RNase H was purchased from Takara Bio Inc. The polymerase used for PCR was the TaKaRa LA Taq HS that was provided the in TaKaRa LA Taq Hot Start Version from Takara Bio Inc. Streptavidin-coated MBs were Dynabeads M-280 purchased from Life Technologies Corporation (Carlsbad, CA, USA). Dynabeads M-280 were washed three times in a buffer containing 10 mM Tris-HCl (pH 7.5) and 2 M KCl before use. Cu(II)APC was purchased from Sigma-Aldrich corporation (St. Louis, MO, USA).

### 3.2. Preparation of Cell Lysate

A pellet of 10^6^ cells was suspended in 200 μL of cold CHAPS lysis buffer provided in the TRAPEZE telomerase detection kit. The cell lysate solution was dispensed into small volume aliquots and stored at −80 °C. Each cell lysate aliquot was diluted in cold CHAPS lysis buffer as appropriate before use.

### 3.3. CPT for MSTP Detection

CPT for MSTP detection was performed to identify a probe RNA suitable for the A-PCR/CPT assay. Each type of probe RNA (100 nM) was annealed separately with each of several concentrations of MSTP in a buffer containing 50 mM Tris-HCl (pH8) and 4 mM MgCl_2_. Each reaction solution was incubated with 0.1 U/μL RNaseH at 30 °C for 0.5 h, and then mixed with 50 mM Na_2_EDTA to terminate the reaction; a fluorescence spectral scanning reader (Varioskan flash; Thermo Fisher Scientific Inc., Waltham, MA, USA) with excitation set to 482 nm was then used to measure the fluorescence intensity of the solution at 500–550 nm and 25 °C.

### 3.4. Electrophoresis Analysis of MSTP

Solutions (10 μL) containing 0.1–10^3^ fmol of MSTP were analyzed by native gel electrophoresis on a 10% nondenaturing polyacrylamide gel in Tris-borate-EDTA buffer (pH 8.5) run at 400 V. The gels were stained with GelStar nucleic acid gel stain and imaged using a fluorescent image analyzer (FLA-5100; Fujifilm Corporation, Tokyo, Japan).

### 3.5. MSTP Digestion Assay

To assess DNase activity in the HeLa cells lysates, HeLa cells lysate was incubated with MSTP, and subject to a digestion assay. MSTP (100 nM) was incubated at 37 °C for 60 min with HeLa cell lysate solution containing 0 or 125 cells/μL in a buffer of 20 mM Tris-HCl (pH 8.0) and 4 mM MgCl_2_ or 10 mM Na_2_EDTA. Each sample was then analyzed *via* gel electrophoresis on a 10% denaturing urea polyacrylamide gel in Tris-borate-EDTA buffer (pH 8.5) at 400 V. Each gel was stained with SYBR Gold nucleic acid gel stain (Life Technologies Corporation) and imaged using a fluorescence image analyzer (FLA-5100).

### 3.6. Telomerase Reaction

Each telomerase reaction solution (10 μL) contained 1×TRAP buffer (TRAPEZE telomerase detection kit), 1×dNTPs (TRAPEZE telomerase detection kit), non-modified TS primer (TRAPEZE telomerase detection kit), HeLa cells lysate, and one of several concentrations of λ DNA; each mixture was incubated at 37 °C for 60 min, heated at 95 °C for 10 min, and cooled at 4 °C. To generate telomerase reaction products that could be immobilized on MBs, 1 μM biotinylated TS primer was used instead of the non-modified TS primer.

### 3.7. Immobilization of Telomerase Reaction Products on MBs

A vortex was used to mix 10 μL of telomerase reaction product, which was generated with 1 μM biotinylated TS primer, with prewashed MBs (Dynabeads M-280) that were in 10 μL of a buffer containing 10 mM Tris-HCl (pH 7.5) and 2 M KCl; these 20 μL were mixed at 25 °C for 30 min. The treated MBs were then subject to three sequential washes, each with 20 μL of buffer containing 10 mM Tris-HCl (pH 7.5) and 1 M NaCl, and one last wash with 20 μL of water. 

### 3.8. A-PCR Amplification of Telomerase Reaction Products

A-PCR amplification of telomerase reaction products was carried out in a solution containing telomerase reaction products (telomerase reaction solutions or MBs with telomerase reaction products), 1×LA PCR Buffer II (Mg^2+^ plus), 1 × dNTPs, any one of several concentrations of TS primer, 1 μM CX-ext primer, and 0.05 U/μL TaKaRa LA Taq HS, for 30 cycles with each cycle comprising denaturation at 95 °C for 30 s, annealing at 59 °C for 30 s, and extension at 72 °C for 30 s. After A-PCR amplification, the reaction mixtures were cooled and stored at 4 °C.

### 3.9. CPT for Detection of A-PCR Products

A-PCR products were detected by CPT. Each CPT reaction mixture (100 μL) contained A-PCR reaction mixture with products (10 μL), 100 nM probe RNA 2, 50 mM Tris-HCl (pH8), and 4 mM MgCl_2_; each mixture was incubated with 0.1 U/μL RNaseH at 37 °C for 20 min. Na_2_EDTA (50 mM) was added to terminate the reaction; a fluorescence spectral scanning reader (Varioskan flash) set with excitation at 482 nm was used to measure the fluorescence intensity of each solution at 500–550 nm and 25 °C.

### 3.10. Normal PCR Amplification of Telomerase Reaction Products

PCR amplification of telomerase reaction products was carried out in a reaction mixtures that each contained telomerase reaction products, 1×LA PCR Buffer II (Mg^2+^ plus) (Takara Bio Inc.), 1×dNTPs (TRAPEZE telomerase detection kit), TS primer (TRAPEZE telomerase detection kit), Primer Mixture solution (TRAPEZE telomerase detection kit), and 0.05 U/μL TaKaRa LA Taq HS (Takara Bio Inc.); amplification occurred over 30 cycles, each comprising denaturation at 95 °C for 30 s, annealing at 59 °C for 30 s, and extension at 72 °C for 30 s. After PCR amplification, each reaction mixture was cooled and stored at 4 °C. The PCR products were analyzed by native gel electrophoresis on a 10% nondenaturing polyacrylamide gel in Tris-borate-EDTA buffer (pH 8.5) at 400 V. The gels were stained with GelStar nucleic acid gel stain and imaged using a fluorescent image analyzer (FLA-5100).

### 3.11. One-Step TRAP Assay (Conventional TRAP Assay)

Each one-step TRAP assay was carried out in a solution containing 1×TRAP buffer (TRAPEZE telomerase detection kit), 1×dNTPs (TRAPEZE telomerase detection kit), non-modified 1 μM TS primer, Primer Mixture solution (TRAPEZE telomerase detection kit), 0.05 U/μL TaKaRa LA Taq HS (Takara Bio Inc.), and HeLa cells lysate as follows: telomerase reaction for one cycle with incubation at 37 °C for 30 min, and PCR for 30 cycles with denaturation at 95 °C for 30 s, annealing at 59 °C for 30 s, and extension at 72 °C for 30 s; after each complete series of cycles, the solution was cooled at 4 °C. The one-step TRAP products were analyzed by native gel electrophoresis on a 10% nondenaturing polyacrylamide gel in Tris-borate-EDTA buffer (pH 8.5) at 400 V. The gels were stained with GelStar nucleic acid gel stain and imaged using a fluorescent image analyzer (FLA-5100).

## 4. Conclusions

An assay for telomerase activity based on A-PCR on MBs and CPT with probe RNA and RNase H was described and tested. The assay allowed the sensitive and selective detection of HeLa cells with a detection limit of 50 HeLa cells although even 10,000 NHDF cells were not detected. This assay also eliminated the false-negative results caused by PCR inhibitors such as bile salt, heparin, and hemoglobin. Furthermore, this assay showed that Cu(II)APC inhibited the telomerase activity of HeLa cells with an IC50 value of 2.2 ± 0.5 μM, indicating that this assay is appropriate for evaluating telomerase inhibition *via* G-quadruplex ligands. Thus, the present assay should be useful for not only cancer diagnosis, but also for screening of anticancer drugs.

## Supplementary Materials

Supplementary File 1
