# Supplementary material for: A Highly Sensitive Telomerase Activity Assay that Eliminates False-Negative Results Caused by PCR Inhibitors
Source: Molecules. 2013 Sep 25;18(10):11751–67. doi: 10.3390/molecules181011751 (PMC6269933; doi:10.3390/molecules181011751)

Supporting Information

Highly Sensitive Telomerase Activity Assay that Eliminates False-negative Results Caused by PCR Inhibitors

Hidenobu Yaku [1,2,3], Takashi Murashima [2,3], Daisuke Miyoshi [2,3,*] and Naoki Sugimoto [2,3,*]

[1] Advanced Technology Research Laboratories, Panasonic Corporation, 3-4 Hikaridai, Seika-cho, Soraku-gun, Kyoto 619-0237, Japan
E-mail: yaku.hidenobu@jp.panasonic.com

[2] Frontier Institute for Biomolecular Engineering Research (FIBER), Konan University, 7-1-20 Minatojima-minamimachi, Chuo-ku, Kobe 650-0047, Japan;
E-mail: murasima@konan-u.ac.jp (T.M.)

[3] Faculty of Frontiers of Innovative Research in Science and Technology (FIRST), Konan University, 7-1-20 Minatojima-minamimachi, Chuo-ku, Kobe 650-0047, Japan

***** Author to whom correspondence should be addressed; For D.M.: Tel: +81-78-303-1426; Fax: +81-78-303-1495; E-mail: miyoshi@center.konan-u.ac.jp. For N.S.: Tel: +81-78-303-1457; Fax: +81-78-303-1495; E-mail: sugimoto@konan-u.ac.jp.

Contents

1. Experimental procedure
2. Supplementary figure

1. Experimental

Electrophoresis analysis of MSTP

Solutions (10 μL) containing 0.1-10^3 fmol of MSTP were analyzed by native gel electrophoresis on a 10% nondenaturing polyacrylamide gel in Tris-borate-EDTA buffer (pH 8.5) run at 400 V. The gels were stained with GelStar nucleic acid gel stain and imaged using a fluorescent image analyzer (FLA-5100).

2. Supplementary Figure

Figure S1. (A) Electrophoresis results of five different amounts of MSTP. (B) Relationship between band intensity of and amount of MSTP.

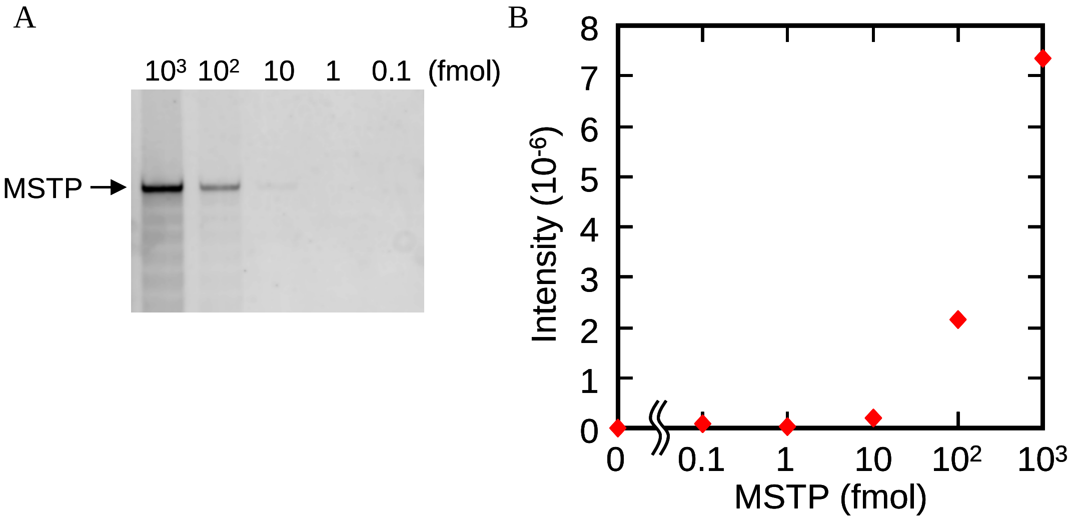

Supplement: Supplementary File 1 [file molecules-18-11751-s001.pdf]
